# Prosodic marking of contrastive focus in Mandarin-speaking children with autism spectrum disorder

**DOI:** 10.3389/fpsyg.2026.1898035

**Published:** 2026-07-24

**Authors:** Jinting Yan, Chen Kuang, Xiao Liang, Xinquan Sun, Fei Chen

**Affiliations:** 1 Institute of Children’s Language Rehabilitation, Cangzhou Normal University, Cangzhou, China; 2 School of Foreign Languages, Hunan University, Changsha, China; 3 School of Foreign Studies, Hunan University of Technology and Business, Changsha, China; 4 Department of Child Rehabilitation, Cangzhou Maternal and Child Health Hospital, Cangzhou, China

**Keywords:** autism spectrum disorder, duration, contrastive focus, intensity, Mandarin Chinese, pitch, post-focus compression, prosodic focus

## Abstract

**Introduction:**

Prosodic focus marking plays a central role in conveying information structure, yet little is known about how Mandarin-speaking children with autism spectrum disorder (ASD) use acoustic cues to signal contrastive focus in a tonal language.

**Methods:**

Prosodic focus marking plays a central role in conveying information structure, yet little is known about how Mandarin-speaking children with autism spectrum disorder (ASD) use acoustic cues to signal contrastive focus in a tonal language. This study examined pitch, duration, and intensity in sentence-initial and sentence-final focus produced by 20 Mandarin-speaking children with ASD and 20 age- and sex-matched typically developing (TD) peers. Using picture-based prompts, children produced simple subject-verb-object sentences, and acoustic measures of focus words (sentence-initial subject and sentence-final object) and post-focus verbs were analyzed with linear mixed-effects models.

**Results:**

Children with ASD showed significantly higher pitch than TD children in sentence-final focus, with no group difference in sentence-initial focus. Duration ratio was significantly reduced only with high-tone verbs. Intensity ratio was lower in sentence-initial focus, but higher in sentence-final focus with high-tone verbs. In the post-focus region, children with ASD exhibited higher pitch than TD children for both high- and low-tone verbs, but showed larger duration ratio and lower intensity ratio only for low-tone verbs.

**Discussion:**

These findings suggest that children with ASD are not globally impaired in focus marking, but show patterns that vary with focus position and tonal context.

## 1 Introduction

Focus marks the central locus of information transmission in an utterance and corresponds to the constituent intended by the speaker to attract the listeners’ primary attention (Halliday, 1967; Xu, 1999). Focus lends itself to multiple taxonomies. In line with Fang (1995), the current study adopts the binary division between natural focus and contrastive focus. Such a dichotomy delineates a core divergence in prosodic realization: natural focus emerges under default information-structural configurations, while contrastive focus is a marked option deliberately used for contrastive purposes to highlight a constituent that is already given in the discourse or mutually known in the context.

Natural focus, also referred to as default focus in some studies, means that in the absence of special pragmatic or syntactic factors, the focus of an utterance in Chinese generally occurs at the end of the sentence (Xu, 2004). This observation is consistent with the end-focus principle, according to which new or salient information tends to appear in sentence-final position.

Contrastive focus occurs when a speaker intends to contrast or correct particular information, allowing focus to appear in different positions within a sentence (Fang, 1995). Whereas natural focus primarily introduces new information, contrastive focus highlights selected information and may be realized through prosodic prominence, syntactic devices, or both (Ge et al., 2023).

Many studies have examined the prosodic features of focus in neurotypical speakers of Mandarin Chinese, focusing primarily on pitch and duration. These studies consistently show that focus is marked by raised pitch and longer duration (on-focus expansion); the pitch of pre-focus elements remains largely unchanged, while post-focus elements are lowered, creating pitch range compression (Xu, 1999; Xu et al., 2012; Chen et al., 2014).

Some researchers have explored how the position of focus affects its prosodic realization. Xu (1999, 2005) reported that pitch is higher when focus occurs at the beginning or middle of a sentence than at the end. Chen and Shi (2011) found that the tonal range of focused words expands at different focus positions. Wang and Xu (2017) studied the impact of focus position and boundary strength on pitch compression and found that post-focus compression can occur across all boundary levels. Wang et al. (2019) further observed that sentence-final focus is typically not followed by pitch compression, which may reduce perceptual accuracy. Overall, existing literature confirms systematic positional variation in the prosodic encoding of focus, particularly in the manifestation of on-focus pitch expansion and post-focus compression.

A body of experimental evidence reveals that prosodic focus modulates Mandarin lexical tones in a tone-specific, position-dependent fashion. Xu (1999) found that focus expands the pitch range of all four Mandarin tones: high targets of high-level, rising, and falling tones increase, and low targets of rising, dipping, and falling tones decrease. The degree of pitch change also depends on focus position, with stronger effects at sentence-initial and sentence-medial positions compared to sentence-final positions. Jia et al. (2006, 2007) showed that focus significantly raises the high tones in disyllabic and five-syllable sequences, while low tones are less affected. Lin (2011) reported that when focus falls on high-level, rising, or falling tones, syllable duration often increases, and duration always increases for dipping-tone syllables. Taken together, these findings converge on the generalization that focus modulates lexical tone contours differentially contingent upon intrinsic tone type, syllabic composition and focus location within an utterance. Previous research has largely centered on how the tone of the focal word itself interacts with focal prosody. The present study builds on this foundation by investigating the role of tonal variation in adjacent non-focal verbs in modulating prosodic focus marking, thus offering a broader perspective on the interplay between lexical tone and focus prosody.

Typically developing (TD) individuals are capable of deploying prosodic focus to mark information prominence, whereas clinical populations such as individuals with autism spectrum disorder (ASD) may exhibit deficits or atypicalities in this prosodic function. Early research on prosodic focus in children with ASD primarily used perceptual methods to determine whether stress was present and where it occurred (Baltaxe, 1984; Fine et al., 1991; Kuang et al., 2026; Shriberg et al., 2001; Paul et al., 2005; Peppé et al., 2007). More recent studies have adopted acoustic measurements to analyze the acoustic features relevant to contrastive focus. For example, van Santen et al. (2010) found equivalent pitch and amplitude for focused targets across ASD and TD children, whereas the ASD group exhibited markedly shorter duration on focused syllables. Nadig and Shaw (2012) reported that although both ASD and TD groups increased pitch, amplitude, and duration when marking contrastive stress, children with high-functioning ASD used adjectives inconsistently and relied less on amplitude than TD children. DePape et al. (2012) suggested that the ability to use focus marking in ASD could be influenced by language skills. Compared with healthy adults, adults with ASD and high language functioning overall employed a wider pitch range but did not mark information structure; by contrast, adults with ASD and moderate language functioning generally used a narrower pitch range, yet were able to mark information structure appropriately to a large extent. Chen et al. (2024) found that children with ASD displayed weaker pitch variation, intensity, and duration in focus stress, particularly for sentence-initial focus. Wang et al. (2024) compared focus-marking performance between Cantonese-speaking and English-speaking children, finding that Cantonese-speaking TD children employed a greater number of acoustic cues for post-focus compression than did Cantonese-speaking ASD children, whereas native English-speaking children exhibited significant differences from the two Cantonese-speaking groups in on-focus expansion. Notably, previous studies on focus production in children with ASD have rarely considered the potential influence of individual differences.

Given the heterogeneous nature of autism, understanding how individual factors contribute to prosodic abilities is of critical importance. Indeed, a recent meta-analysis identified language abilities and nonverbal IQ as effective modulators of prosodic focus production (Kuang et al., 2026). Furthermore, working memory has been widely recognized for its essential role in language acquisition and online linguistic processing (Baddeley, 2000; Gathercole and Baddeley, 2014) and has been frequently examined for its effects on prosodic abilities in children with ASD (e.g., Ge et al., 2023; Wang et al., 2023). Thus, the present study also investigates whether individual differences in language, working memory, and nonverbal IQ affect focus production in children with ASD.

To conclude, previous studies indicate that children with ASD may encounter difficulties in expressing contrastive focus. Nevertheless, existing findings have been inconsistent, which may be attributed to variations in research methodologies, participant characteristics, and language backgrounds. Most previous studies have focused on English or other non-tonal languages, and systematic investigations into prosodic focus among Mandarin-speaking children with ASD remain scarce. Accordingly, the present study aims to systematically examine the prosodic features of contrastive focus in Mandarin-speaking children with ASD.

Given that Mandarin is a tone language in which pitch simultaneously conveys lexical and post-lexical (i.e., focus-related) information, it remains an open question whether Mandarin-speaking children with ASD exhibit similar or distinct patterns of focus prosody compared to their TD peers. Moreover, the potential modulating roles of focus position (sentence-initial subject vs. sentence-final object) and lexical tone (tone patterns of verbs) have not been systematically examined in this population.

To address these gaps, the current study aims to investigate the prosodic characteristics of contrastive focus in Mandarin-speaking children with ASD, with a particular focus on pitch, duration, and intensity. Specifically, we ask: (1) Whether the acoustic realization of contrastive focus (pitch, duration, and intensity) differs between Mandarin-speaking children with ASD and age-matched TD children; (2) Whether focus position (sentence-initial vs. sentence-final) modulates the prosodic marking of contrastive focus differently in the two groups; and (3) Whether the lexical tone of adjacent non-focal verbs (high-tone vs. low-tone) differentially influences the prosodic realization of contrastive focus in children with ASD and TD children.

## 2 Materials and methods

### 2.1 Participants

All Mandarin-speaking children with ASD were recruited from the Cangzhou Research Center for Child Language Rehabilitation. The clinical diagnosis of autism was based on the criteria outlined in the Diagnostic and Statistical Manual of Mental Disorders, Fifth Edition (DSM-5; American Psychiatric Association, 2013), and was further confirmed by pediatricians from Grade-A tertiary hospitals using the Autism Behavior Checklist (ABC; Krug et al., 1978, 1980) and the Childhood Autism Rating Scale (CARS; Schopler and Reichler, 1980, 1990). For comparative analysis, 20 typically developing (TD) children were selected as the control group.

In our sample, TD children (18 boys and 2 girls) ranged from 5.5 to 10.5 years (66–126 months), and ASD children (18 boys and 2 girls) ranged from 5.7 to 9.7 years (68–116 months). The two groups were statistically matched for chronological age and sex but differed significantly in working memory, language ability, and nonverbal intelligence (all *p*s < 0.001). This study was approved by the Research Ethics Committee of Cangzhou Normal University, and informed consent was obtained from the parents or legal guardians of all participants.

Children’s language ability was assessed in detail using five subtests from the Test of Language Ability in Mandarin-Speaking Preschoolers, which included the Test of Mandarin Grammar, Word Definition Test, Rapid Automatized Naming, Narrative Test, and Sentence Comprehension Test (Ning, 2013). These subtests together provided a comprehensive evaluation of children’s language skills, covering phonological awareness, vocabulary knowledge, syntactic understanding, and semantic processing. Each subtest offered insight into a different aspect of language ability, allowing for a nuanced understanding of individual strengths and weaknesses.

Working memory was measured using a digit-span task. In this task, participants were asked to verbally repeat sequences of digits presented to them either in the same order as given (forward digit span) or in reverse order (backward digit span). The task was discontinued once a participant failed two trials of the same length, ensuring that the assessment captured the maximum span the child could accurately handle. Scores were calculated based on the number of correct trials, with a maximum total of 32 points—16 for the forward span and 16 for the backward span. This method allowed for a detailed measurement of short-term and working memory capacities.

Nonverbal intelligence was assessed using the Primary Test of Nonverbal Intelligence (PTONI; Ehrler and McGhee, 2008), a well-established, research-based instrument designed to measure reasoning and problem-solving abilities in young children aged between 3;0 and 9;11 years. The PTONI uses nonverbal tasks to assess cognitive abilities independent of language skills, providing a reliable indication of a child’s general reasoning capacity. By combining assessments of language, working memory, and nonverbal intelligence, this study was able to account for multiple cognitive dimensions that could influence prosodic focus marking in children with ASD. Participant information is summarized in Table 1.

**Table 1:** Participant demographics and cognitive characteristics.

Characteristics	Children with ASD (*n* = 20)		TD children (*n* = 20)		*t*	*p*
	Mean	SD	Mean	SD		
Age (months)	94.09	20.50	85.39	14.63	1.55	0.129
Working memory	11.45	6.19	18.30	3.21	−4.38	<0.001
Language ability	69.50	14.52	94.10	4.70	−7.11	<0.001
Nonverbal intelligence	70.55	11.45	103.05	14.84	−7.75	<0.001

### 2.2 Materials

Considering the linguistic and cognitive levels of children with ASD, this study used simple sentences with a subject-verb-object structure. Contrastive focus was elicited in sentence-initial and sentence-final positions. The nouns were high-frequency words commonly used in daily life and covered the four lexical tones of Mandarin Chinese. The monosyllabic verbs included “eat” (*chī*, Tone 1 in Mandarin, high-level tone) and “wash” (*xǐ*, Tone 3 in Mandarin, low dipping tone), allowing us to examine how high- and low-tone verbs affect focus prominence. The target sentences are presented in Table 2.

**Table 2:** Target sentences used in the experiment.

Subject	Verb	Object
Rooster/ox/puppy/elephant/kitten (剬鸡/黄牛/小狗/大象/小猫)	Eats/washes (吃/洗)	Bananas/blueberries/purple sweet potatoes/mung beans/strawberries (香蕉/蓝莓/紫薯/绿豆/草莓)

To make the experiment more engaging for children, all sentences were paired with vivid illustrations (50 in total) and presented in a randomized order. Of the 50 pictures, 25 were paired with high-tone verbs and 25 with low-tone verbs, evenly distributed across sentence-initial and sentence-final focus conditions. Each slide displayed, on the left, a non-experimental sentence pre-recorded by a student specializing in broadcasting and hosting, and, on the right, the experimental sentence that child participants were asked to produce.

### 2.3 Procedure

Recordings were conducted in a sound-attenuated booth. First, the children familiarized themselves with the words used in the experiment. They then learned the specific experimental procedures, including viewing the PowerPoint slides, listening to pre-recorded prompting sentences, and attempting to produce the target sentence on the right side of each slide. Finally, under the guidance of the experimenter, the children completed recordings of all experimental sentences.

**Figure 1:**
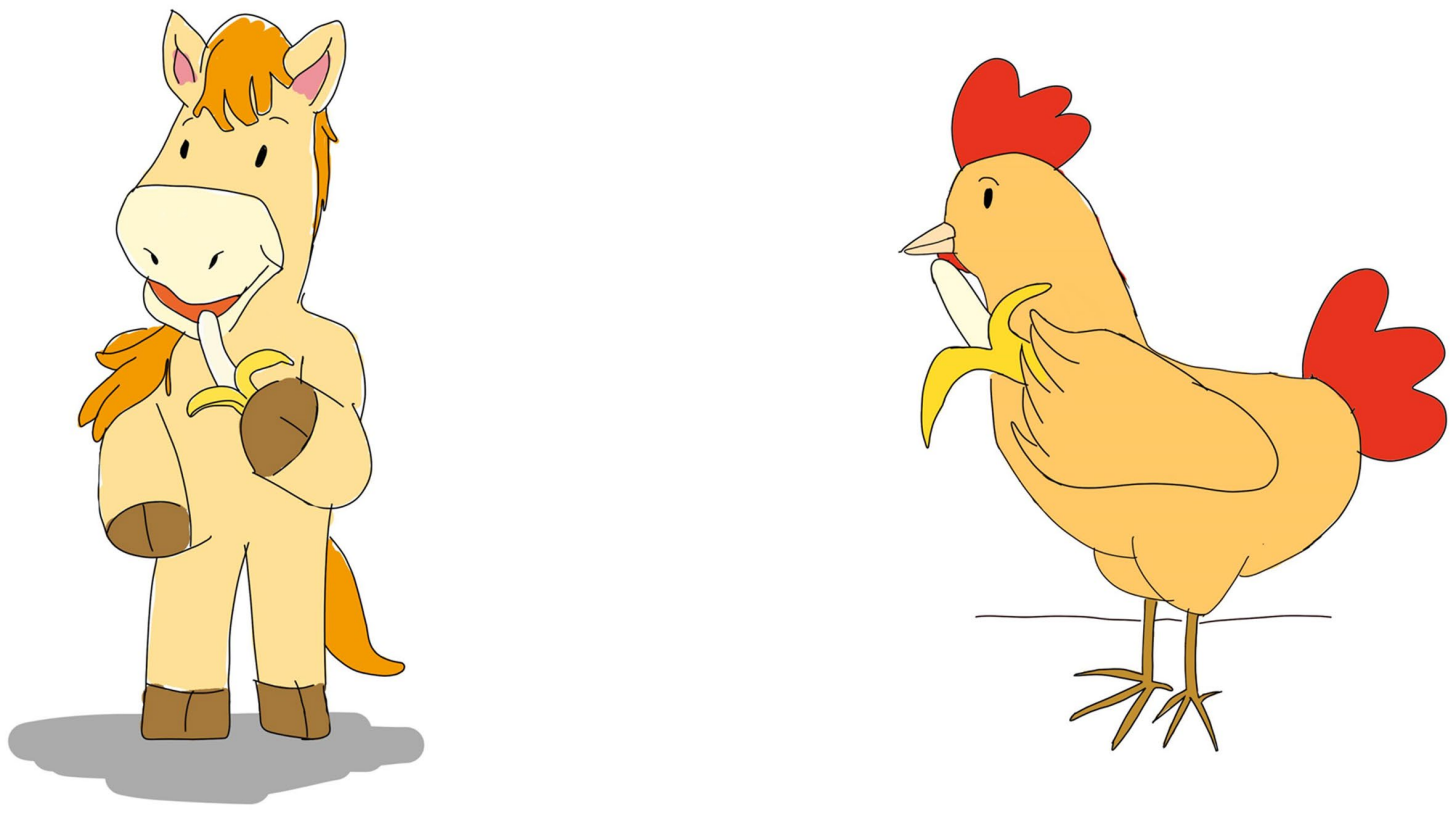
Example of a picture-based prompt for sentence-initial focus.

The recordings included two conditions: sentence-initial focus and sentence-final focus. For sentence-initial focus, as illustrated in Figure 1, the experimenter pointed to the picture on the left and played the recording “The pony eats bananas” (emphasizing “pony”), then asked, “What about this one?” while pointing to the picture on the right. The child responded, “The rooster eats bananas,” correctly placing the stress on “rooster”.

**Figure 2:**
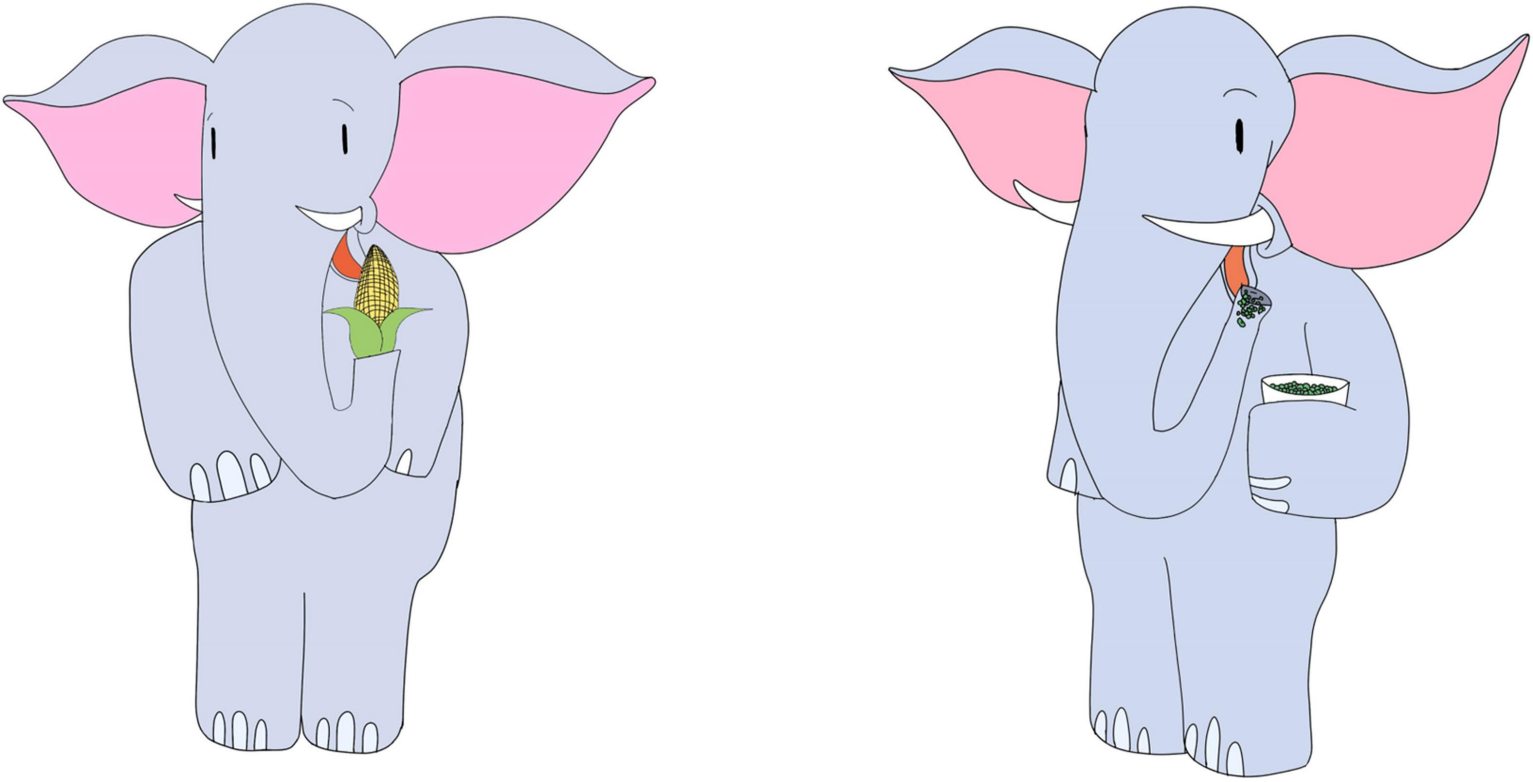
Example of a picture-based prompt for sentence-final focus.

For sentence-final focus, as illustrated in Figure 2, the experimenter pointed to the picture on the left and played the recording “The elephant eats corn” (emphasizing “corn”), then asked, “What about this one?” while pointing to the picture on the right. The child responded, “The elephant eats mung beans,” correctly stressing “mung beans”.

Children’s responses were recorded using Praat software at a 44,100 Hz sampling rate, 16-bit mono. The recording setup included a Takstar PCM-5520 professional condenser microphone and an Avid Mbox 3 external sound card. The Takstar PCM-5520 microphone was positioned at a fixed distance of 20 cm from the child’s mouth, with consistent height and angle maintained across sessions. To address the challenge of posture instability commonly observed in child populations, the experimenter continuously monitored each participant and provided gentle reminders to maintain a steady position throughout the recording. This protocol was implemented to minimize intensity fluctuations arising from variations in recording distance and to reduce potential motion-related acoustic confounds.

### 2.4 Data analysis

The experimental sentences were analyzed acoustically using MinispeechLab, a desktop speech laboratory developed at Nankai University. All acoustic data in this study were processed using MiniSpeechLab software. Before the extraction of fundamental frequency (F0), we manually refined all speech waveforms and F0 contours: abnormal elbow protrusions at the onset of contours and unnatural falling tails at the end of utterances were removed, and irregular breaks and incoherent distortions in waveforms were manually repaired.

In regard to the procedures for F0 correction, we selected the function of “Correct F0 based on narrow-band spectrum” under the “F0-waveform” panel. Segments highlighted in blue on the interface required manual adjustment. We modified the F0 trajectories following the general trend of the red narrow-band spectrum and eliminated abnormal elbows and falling tails consistently. All F0 values were extracted from the fifth harmonic of speech signals and automatically generated by the software after calibration.

We have also adopted standardized operations to remove or repair defective waveform segments. To delete a waveform fragment, we clicked its start and end points with the left mouse button and pressed the “Z” key. For discontinuous broken waveforms, we clicked the break point with the left mouse button and confirmed with the right mouse button to reconnect the split segments. Next, nine equidistant F0 points (in Hz) were extracted from each syllable and converted into semitone values (reference = 100 Hz) using the formula:

$$\text{Semitone}=12\times \log_{2}(F0/100)$$

The reference frequency was set to 100 Hz, which is commonly adopted in child speech analysis (Chen et al., 2022; Rattanasone et al., 2018). Pitch range was calculated as the maximum minus the minimum pitch (in semitones) for the focus word (two syllables) and the post-focus verb (one syllable).

For duration, each syllable’s duration was measured. The duration ratio was calculated as:

$$\mathrm{Dx}=\frac{\mathrm{Sx}}{\text{Smean}}$$

*Sx* is the duration of syllable *x*. *Smean* is the mean syllable duration within the relevant sequence (Shi et al., 2010).

For intensity, the amplitude product of each syllable was measured, and an intensity ratio was calculated as:

$$\mathrm{Ex}=\frac{\mathrm{Gx}}{\text{Gmean}}$$

*Gx* is the amplitude product of a given syllable and *Gmean* is the mean amplitude product per syllable across the sentence (Shi et al., 2010).

Notably, the normalization strategy differed across acoustic measures. Duration and intensity are heavily influenced by intra-sentence prosodic context and utterance length. Relative normalization against the mean value of each sentence was therefore applied to eliminate sentence-level confounding factors, facilitating comparisons of prosodic contrasts between focal and non-focal units within the same sentence. In contrast, raw pitch values show substantial inter-individual physiological variation, and semitone conversion is widely used in the field to offset disparities in speakers’ baseline pitch (Chen et al., 2022; Rattanasone et al., 2018). Since our pitch contour analysis was conducted at the syllable level, we did not apply additional normalization based on sentence means, in order to fully preserve tonal range differences across syllables.

Pitch, duration, and intensity were analyzed using linear mixed-effects models fitted in R (R Core Team, 2025) with the *lme4* package (Bates et al., 2015). When a significant main effect of a multi-level factor or a significant interaction was found, post-hoc pairwise comparisons were conducted using the *emmeans* package (Lenth et al., 2018), with Tukey’s adjustment for multiple comparisons. The reported statistics (*t* or *z*) for pairwise comparisons follow the *emmeans* package’s default output based on the degrees of freedom estimation method. Visualization was performed using ggplot2 (Wickham, 2016). Pitch contours were plotted by connecting the nine normalized points per syllable, with smoothing (method = “gam”) applied for visual presentation.

## 3 Results

### 3.1 Prosodic characteristics of focus words

In the following sections, we analyze the prosodic characteristics of focus words produced by children with ASD when expressing sentence-initial (subject) and sentence-final (object) contrastive focus in terms of pitch, duration, and intensity. Four linear mixed-effects models were constructed to examine the mean pitch, pitch range, duration, and intensity data. “Group” (ASD vs. TD), “Focus Position” (sentence-initial subject vs. sentence-final object), and “Verb Type” (high tone vs. low tone) were regarded as fixed factors. Working memory, language ability, and nonverbal IQ were included as covariates. Random intercepts and slopes were specified for subjects and items.

#### 3.1.1 Pitch of focus words

**Figure 3:**
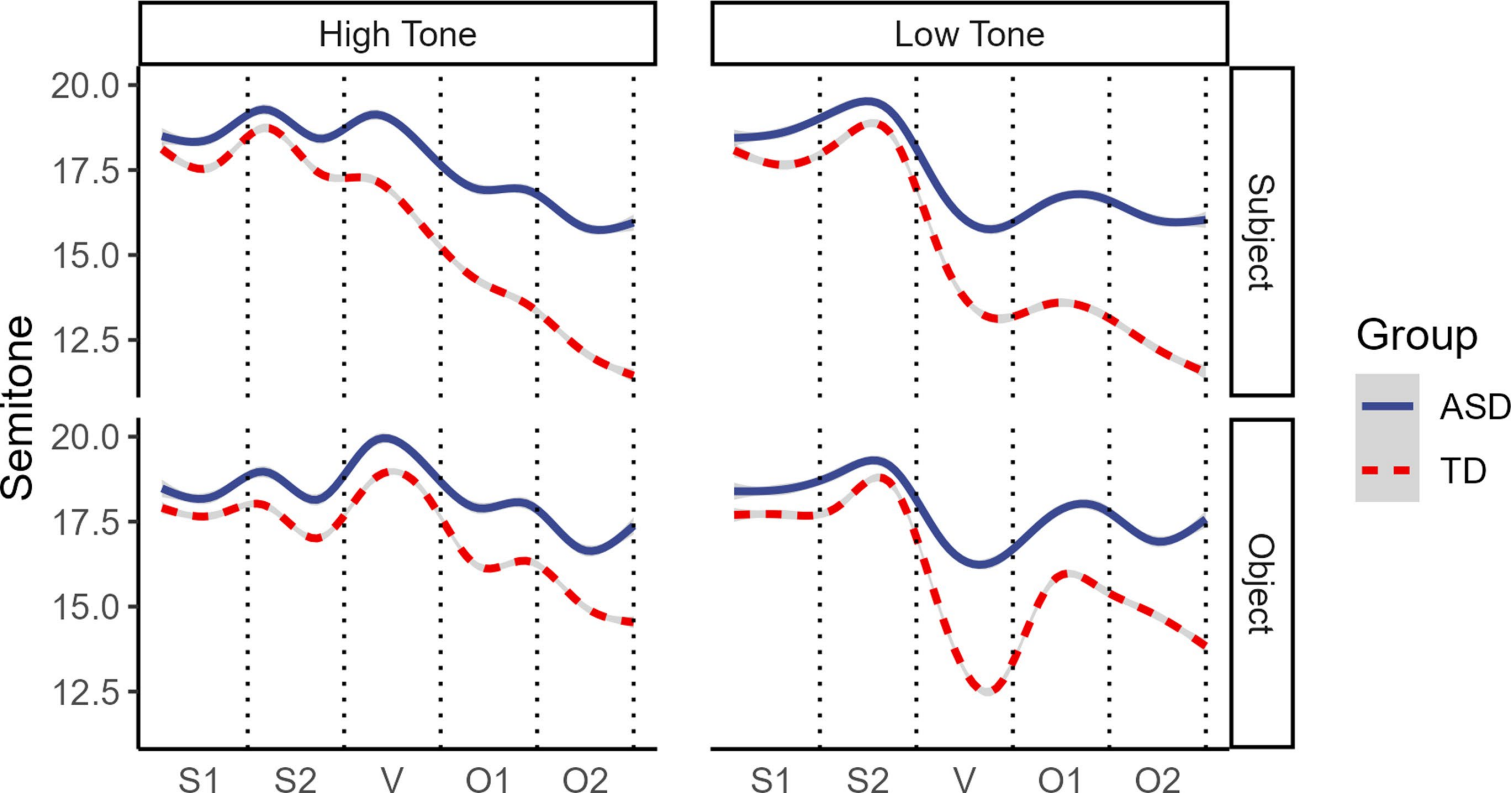
Mean pitch contours of sentence-level pitch for children with ASD and TD children across conditions. The x-axis represents the normalized time sequence across syllables, with vertical dashed lines indicating syllable boundaries (S1, S2 = subject noun; V = verb; O1, O2 = object noun). Pitch values were extracted from each syllable at 9 equidistant time points, converted to semitones (reference = 100 Hz), and concatenated in sequence to form sentence-level contours. Shaded areas represent 95% confidence intervals.

Figure 3 displays the sentence pitch contours, with each syllable positioned between two adjacent vertical dashed lines. Specifically, Figure 3 illustrates the pitch differences between the ASD group (blue solid line) and the TD group (red dashed line). With each syllable divided into 9 equidistant time points, the 45 points from the five syllables were concatenated in sequence to reconstruct the sentence-level contours.

**Figure 4:**
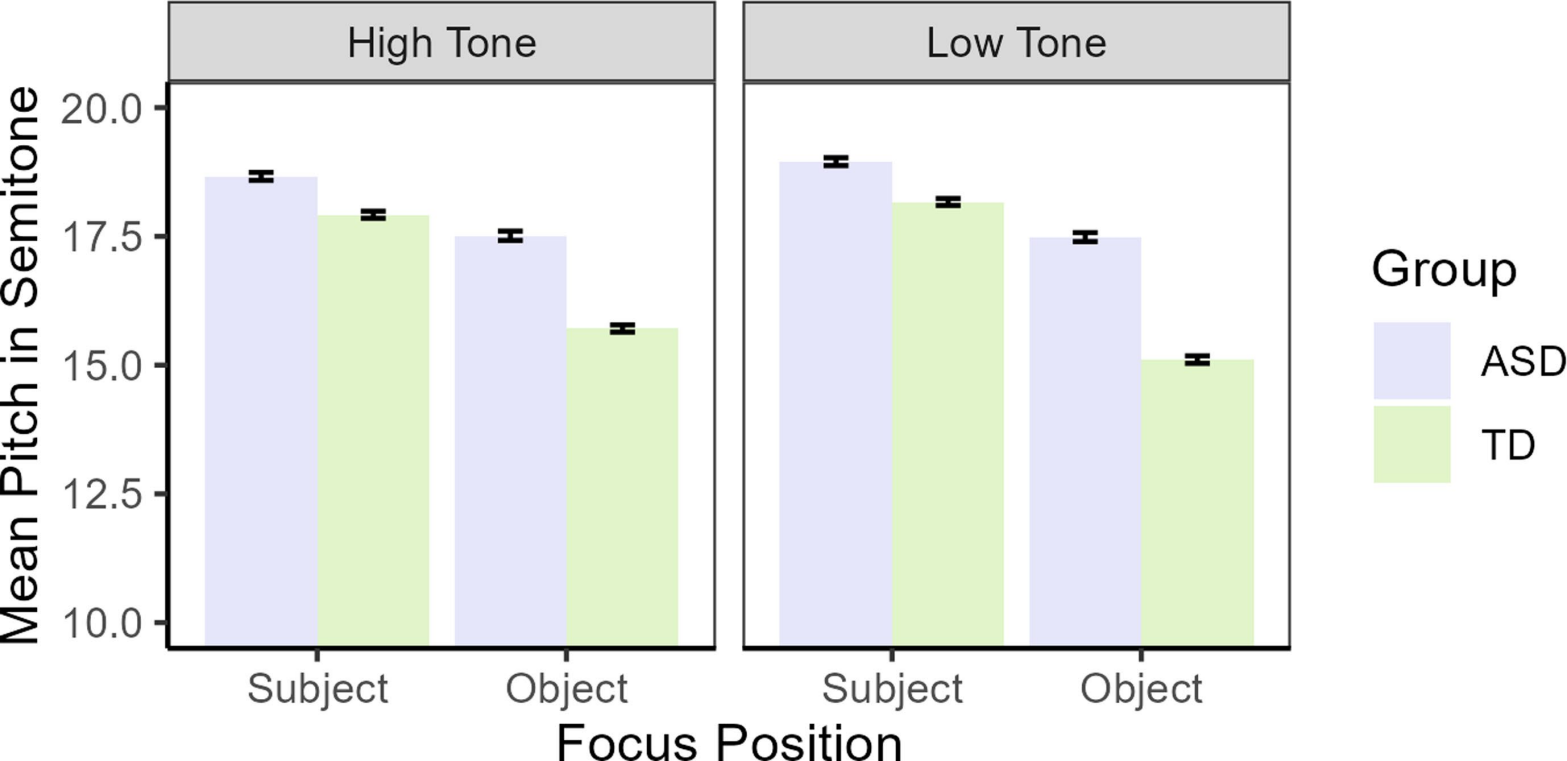
Mean pitch of focus words in children with ASD and TD children.

The results of the mean pitch analysis (see Figure 4) show a significant main effect of “Focus Position” [*χ*^2^ (1) = 32.34, *p* < 0.001] as well as a significant interaction between “Group” and “Focus Position” [*χ*^2^ (1) = 15.78, *p* < 0.001]. Other main effects or interactions did not reach significance (all *p*s > 0.05). Individual-difference factors such as working memory, language level, and nonverbal intelligence were also not found to have significant effects on the pitch of focus words (all *p*s > 0.05).

Further pairwise comparisons on the interaction between “Group” and “Focus Position” revealed that, when the focus was sentence-initial, the pitch-contour difference between the ASD and TD groups did not reach statistical significance (*β* = 1.41, *SE* = 1.20, *z* = 1.17, *p* = 0.241). When the focus was sentence-final, however, the ASD group had significantly higher pitch than the TD group (*β* = 2.68, *SE* = 1.21, *z* = 2.21, *p* = 0.027). Compared with TD children, children with ASD showed distinctive pitch characteristics when expressing sentence-final focus.

**Figure 5:**
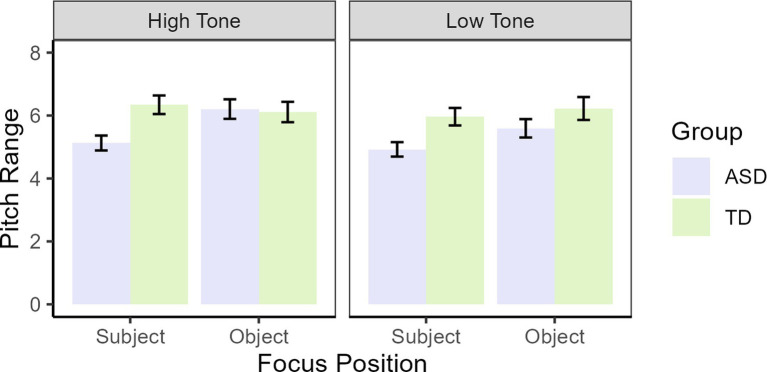
Pitch range of focus words in children with ASD and TD children.

The results of the pitch range analysis (see Figure 5) show a significant interaction between “Group” and “Focus Position” [*χ*^2^ (1) = 4.98, *p* = 0.026]. Other main effects or interactions did not reach significance (all *p*s > 0.05). Individual-difference factors such as working memory, language level, and nonverbal intelligence were also not found to have significant effects on the pitch of focus words (all *p*s > 0.05).

Further pairwise comparisons on the interaction between “Group” and “Focus Position” revealed that, when the focus was sentence-initial, the pitch-contour difference between the ASD and TD groups did not reach statistical significance (*β* = −1.07, *SE* = 0.74, *z* = −1.44, *p* = 0.150). Similarly, when the focus was sentence-final, the pitch range of ASD group did not differ from the TD group (*β* = 0.05, *SE* = 0.71, *z* = 0.07, *p* = 0.945).

#### 3.1.2 Duration of focus words

**Figure 6:**
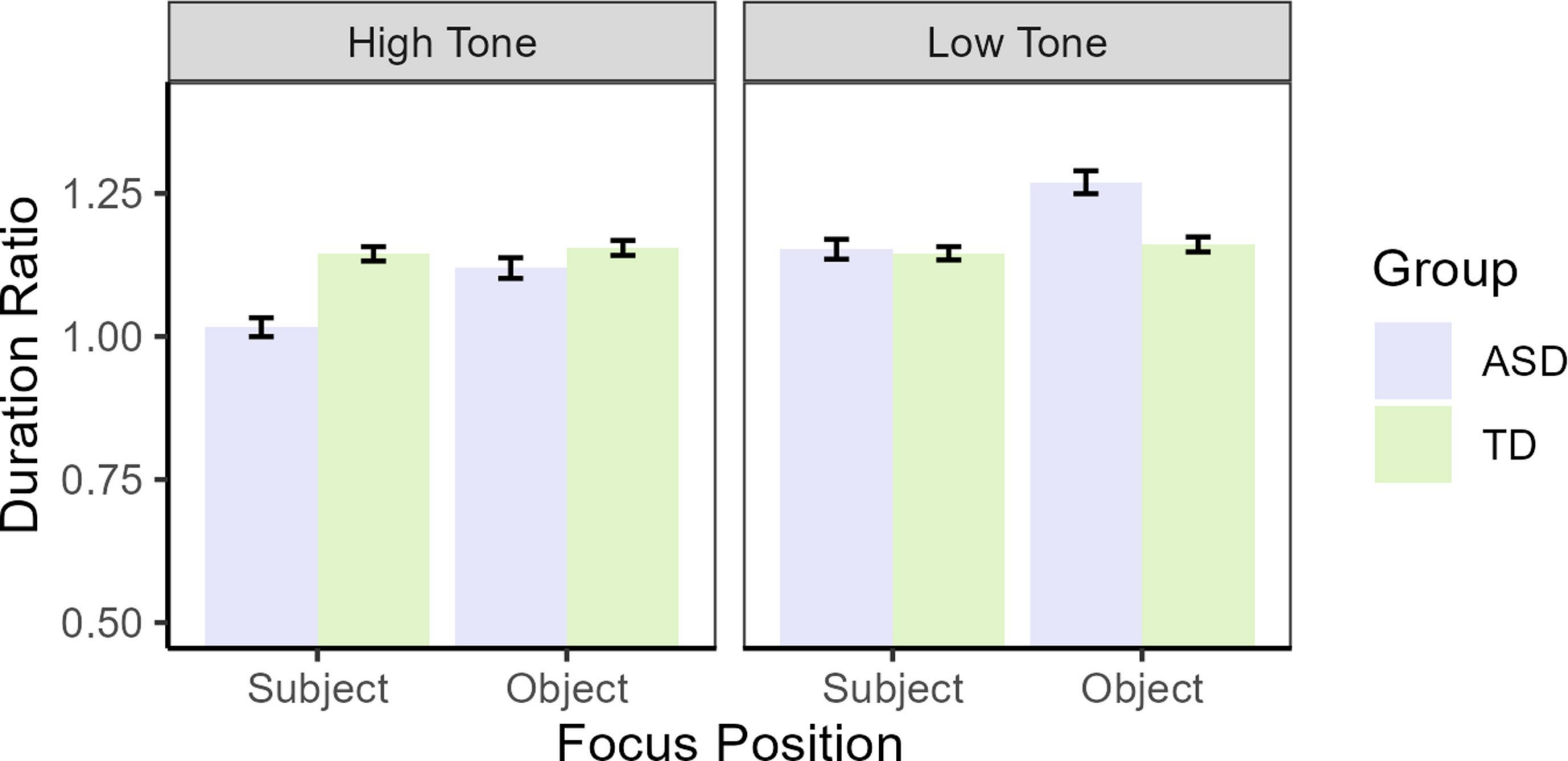
Duration patterns of focus words in children with ASD and TD children.

The results of the duration analysis (see Figure 6) revealed significant main effects of “Focus Position” [*χ*^2^(1) = 5.76, *p* = 0.016], and “Verb Type” [*χ*^2^(1) = 17.97, *p* < 0.001]. Furthermore, there were significant two-way interactions between “Group” and “Focus Position” [*χ*^2^(1) = 5.32, *p* = 0.021], and between “Group” and “Verb Type” [*χ*^2^(1) = 53.62, *p* < 0.001]. No other main effects or interactions reached significance (all *p*s > 0.05). Individual-difference factors such as working memory, language level, and nonverbal intelligence were not found to have significant effects on the duration of focus words (all *p*s > 0.05).

Pairwise comparisons on the interaction between “Group” and “Focus Position” revealed that, the duration ratio of ASD and TD group did not differ in both sentence-initial(*β* = −0.07, *SE* = 0.04, *z* = −1.70, *p* = 0.089) and sentence final conditions (*β* = 0.03, *SE* = 0.04, *z* = 0.682, *p* = 0.495).

Moreover, pairwise comparisons on the interaction between “Group” and “Verb Type” showed that, when the verb was high-toned, the ASD group produced a significantly smaller focus duration ratio than the TD group (*β* = −0.09, *SE* = 0.04, *z* = −2.64, *p* = 0.008). For the low-toned verb, however, the focus duration ratio did not differ significantly between the two groups (*β* = 0.05, *SE* = 0.04, *z* = 1.33, *p* = 0.182).

#### 3.1.3 Intensity of focus words

**Figure 7:**
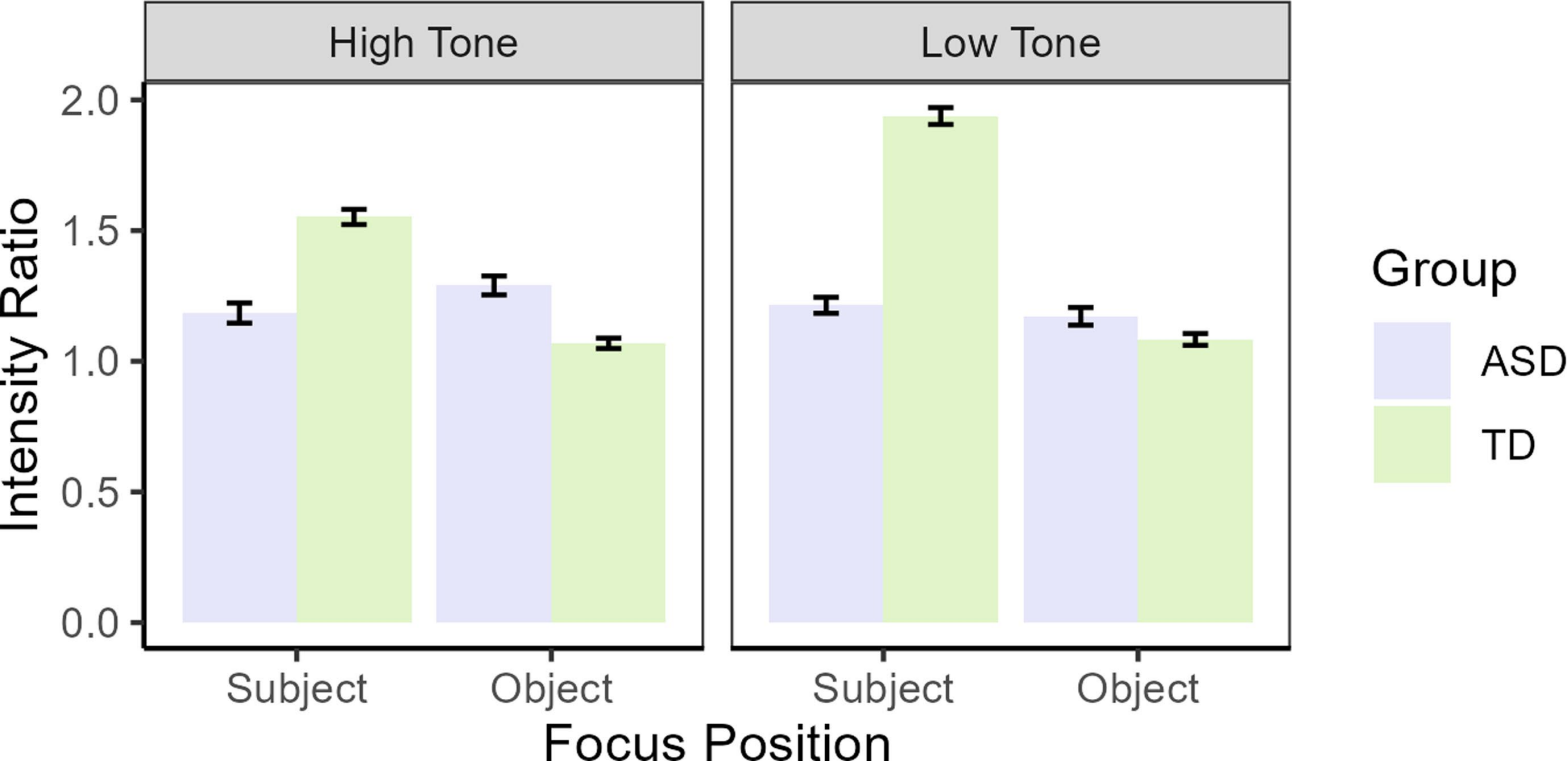
Intensity patterns of focus words in children with ASD and TD children.

The intensity analysis (see Figure 7) yielded significant main effects of “Group” [*χ*^2^(1) = 6.35, *p* = 0.012], “Focus Position” [*χ*^2^(1) = 28.78, *p* < 0.001], and “Verb” [*χ*^2^(1) = 8.54, *p* = 0.003]. However, these main effects were qualified by significant two-way interactions for all pairs. Significant two-way interactions were found between “Group” and “Focus Position” [*χ*^2^(1) = 35.64, *p* < 0.001], “Group” and “Verb Type” [*χ*^2^(1) = 65.84, *p* < 0.001], and “Focus Position” and “Verb Type” [*χ*^2^(1) = 21.97, *p* < 0.001]. Most notably, the three-way interaction a significant three-way interaction among “Group,” “Focus Position,” and “Verb Type” [*χ*^2^ (1) = 17.79, *p* < 0.001], suggesting that the prosodic marking of intensity in focus words differs between groups in a manner that depends jointly on focus position and verb type. None of the covariates were significant (all *p*s > 0.05).

Pairwise comparisons were conducted to decompose the significant three-way interaction among “Group,” “Focus Position,” and “Verb Type.” Under the sentence-initial focus condition, when the verb was high-toned, the intensity ratio of the ASD group was significantly lower than that of the TD group (*β* = −0.33, *SE* = 0.08, *z* = −4.22, *p* < 0.001); when the verb was low-toned, the intensity ratio of the ASD group was also significantly lower than that of the TD group (*β* = −0.69, *SE* = 0.08, *z* = −8.79, *p* < 0.001), further supporting the phenomenon of insufficient intensity in the ASD group under sentence-initial focus. Under the sentence-final focus condition, when the verb was high-toned, the intensity ratio of the ASD group was significantly higher than that of the TD group (*β* = 0.26, *SE* = 0.07, *z* = 3.49, *p* = 0.001); when the verb was low-toned, the difference in intensity ratio between the ASD and TD groups was not significant (*β* = 0.13, *SE* = 0.07, *z* = 1.71, *p* = 0.088).

### 3.2 Prosodic characteristics of post-focus verbs

The prosodic performance of post-focus verbs often contrasts with other sentence components, thereby highlighting the meaning of the focus through a phenomenon known as post-focus compression (Wang and Xu, 2017). In the current analysis, we focus specifically on verbs following subject focus (sentence-initial focus), as only subject focus leaves linguistic material in the post-focus position. For object focus (sentence-final position), there is no subsequent content following the focus, making post-focus compression unmeasurable. Therefore, the analysis of post-focus verbs is necessarily limited to the subject focus condition. Analyzing this contrast can deepen our understanding of the realization of focus in children with ASD. Four linear mixed-effects models were constructed to analyze mean pitch, pitch range, duration, and intensity. “Group” (ASD vs. TD) and “Verb Type” (high tone vs. low tone) were entered as fixed factors, with working memory, language ability, and nonverbal IQ as covariates. Random intercepts and slopes were specified for subjects and items.

#### 3.2.1 Pitch of post-focus verbs

**Figure 8:**
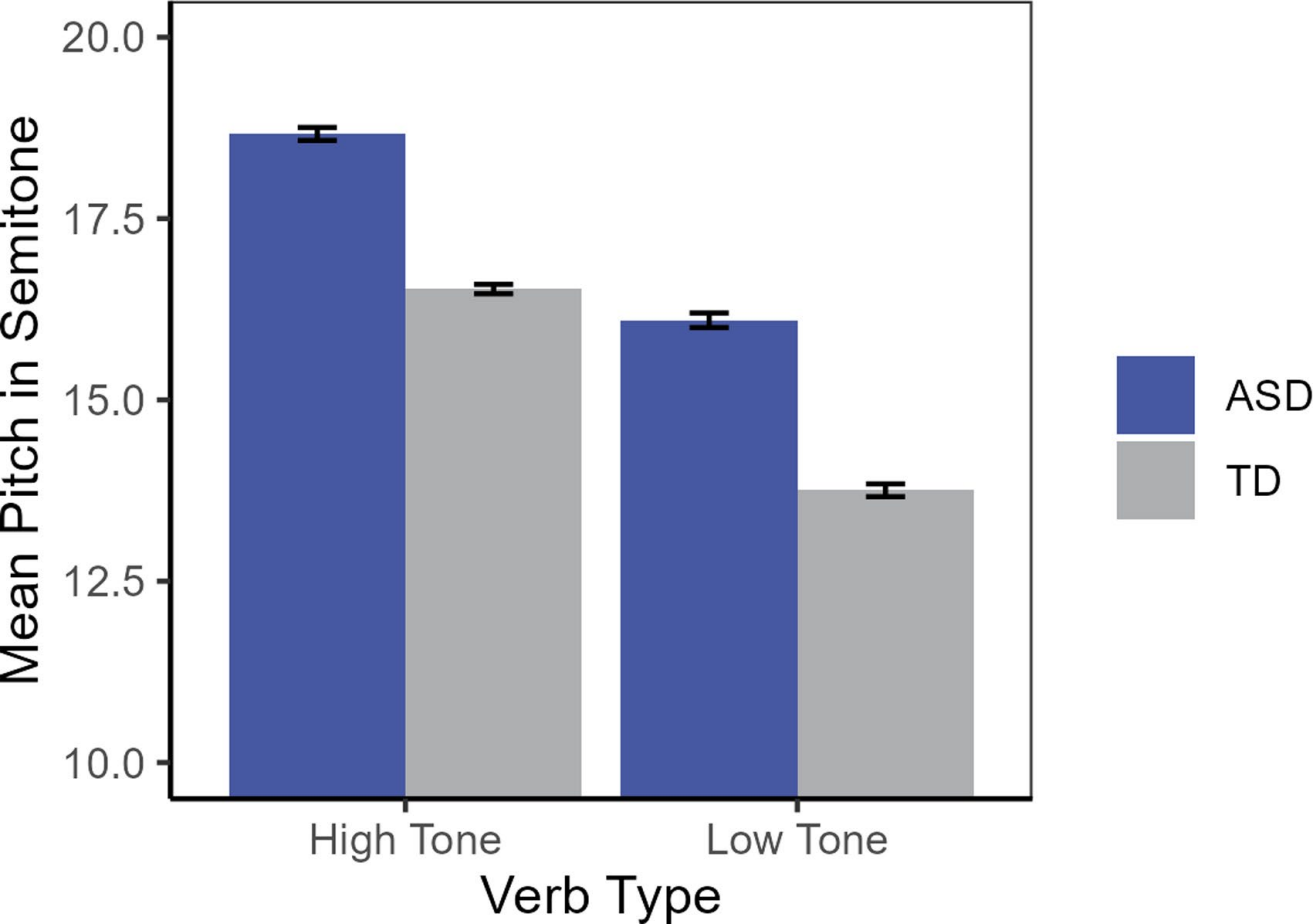
Mean pitch of post-focus verbs in children with ASD and TD children.

The pitch results for post-focus verbs (see Figure 8) show significant main effects of “Group” [*χ*^2^(1) = 4.77, *p* = 0.029] and “Verb Type” [*χ*^2^(1) = 53.58, *p* < 0.001]. No significant interaction was found between “Group” and “Verb Type” (*p* > 0.05). Notably, nonverbal intelligence had a significant effect on the pitch of post-focus verbs (*β* = 1.09, *SE* = 0.50, *t* = 2.17, *p* = 0.036), the higher the level of nonverbal intelligence, the higher the verb pitch. Language level and working memory did not show significant effects on verb pitch (all *p*s > 0.05).

Post-hoc pairwise comparisons on the main effect of “Group” showed that verb pitch in the ASD group was significantly higher than that in the TD group (*β* = 2.41, *SE* = 1.09, *z* = 2.21, *p* = 0.027). Similarly, post-hoc comparisons on the main effect of “Verb Type” revealed that high-toned verbs produced significantly higher pitch than low-toned verbs (*β* = 2.66, *SE* = 0.30, *z* = 8.88, *p* < 0.001).

**Figure 9:**
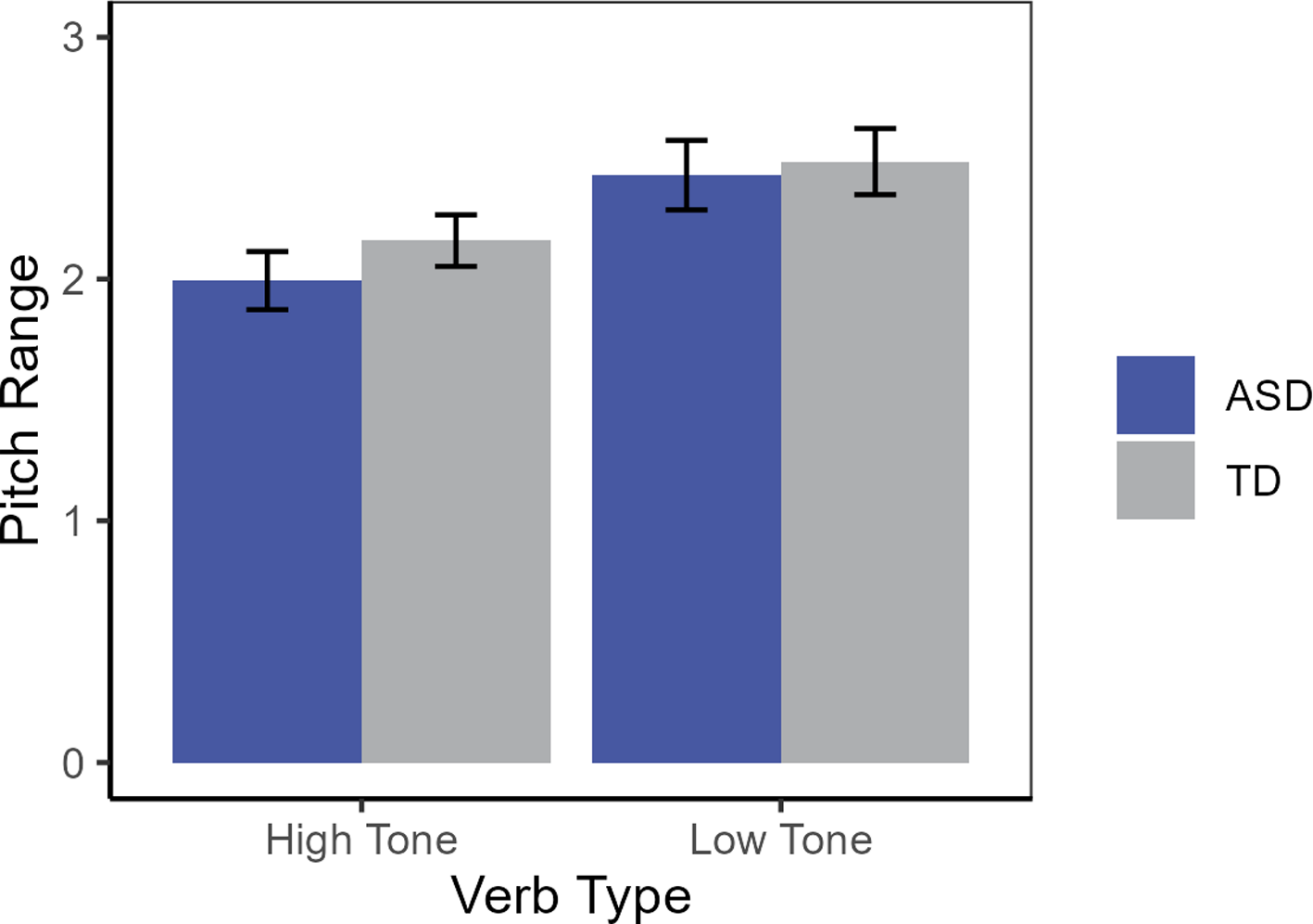
Pitch range of post-focus verbs in children with ASD and TD children.

The results of the pitch range analysis (see Figure 9) show a significant main effect of “Verb Type” [*χ*^2^ (1) = 4.92, *p* = 0.027]. Other main effects or interactions did not reach significance (all *p*s > 0.05). Individual-difference factors such as working memory, language level, and nonverbal intelligence were also not found to have significant effects on the pitch of focus words (all *p*s > 0.05).

Further pairwise comparisons were conducted to examine the main effect of “Verb Type.” The results revealed that low-toned verbs showed larger pitch range than high-toned verbs (*β* = −0.40, *SE* = 0.17, *z* = −2.43, *p* = 0.018).

#### 3.2.2 Duration of post-focus verbs

**Figure 10:**
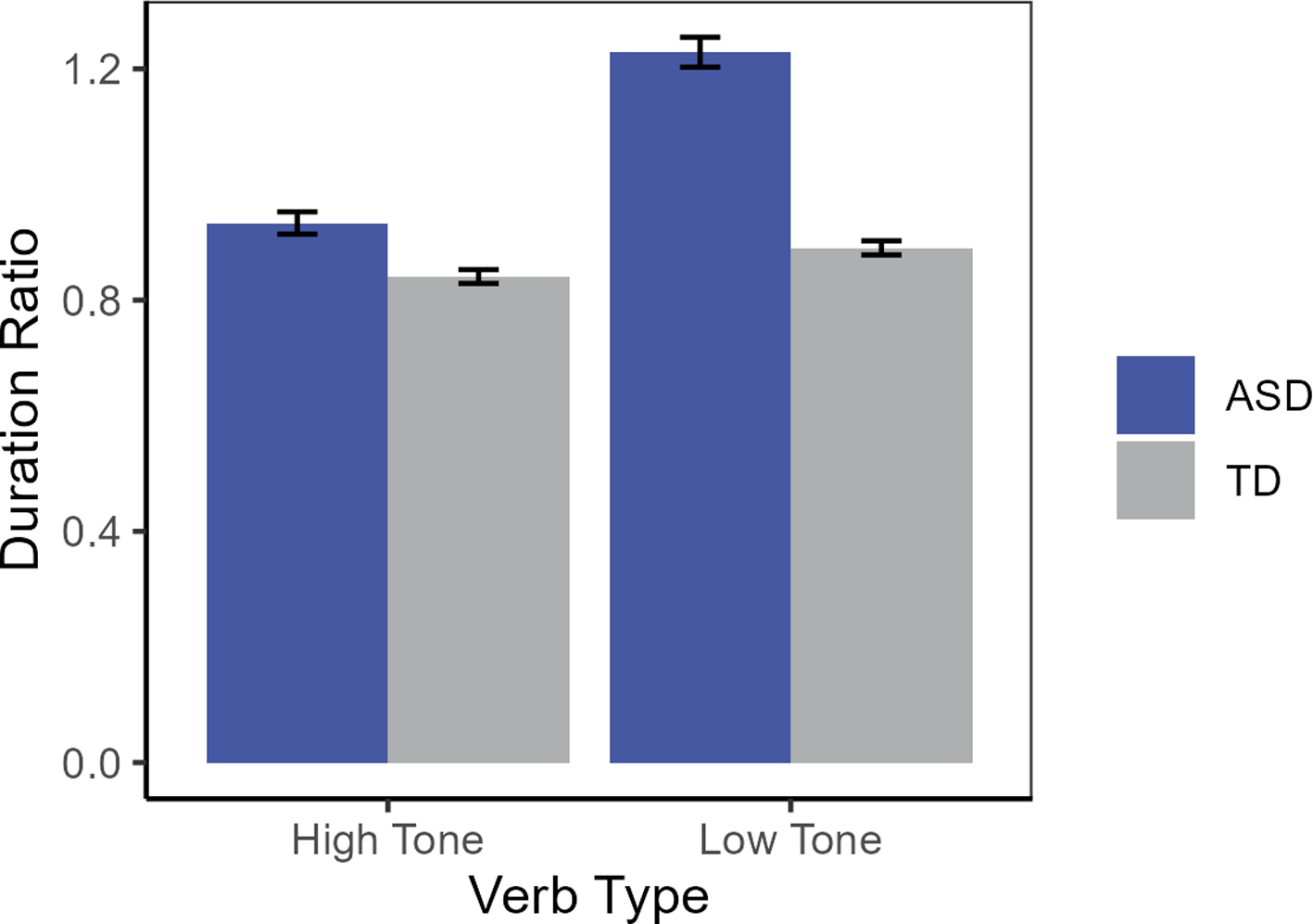
Duration patterns of post-focus verbs in children with ASD and TD children.

The duration results for post-focus verbs (see Figure 10) revealed significant main effects of “Group” [*χ*^2^(1) = 11.93, *p* < 0.001] and “Verb Type” [*χ*^2^(1) = 30.41, *p* < 0.001]. Moreover, we found a significant interaction between “Group” and “Verb Type” [*χ*^2^(1) = 26.26, *p* < 0.001]. Notably, working memory, language level, and nonverbal intelligence did not show significant effects on the duration ratio of verbs following sentence-initial focus (all *p*s > 0.05).

Pairwise comparisons on the interaction between “Group” and “Verb Type” revealed that, for high-tone verbs, the duration ratio difference in post-focus verbs between the ASD and TD groups was not significant (*β* = 0.08, *SE* = 0.06, *t* = 1.38, *p* = 0.175), whereas for low-tone verbs, the duration ratio of post-focus verbs in the ASD group was significantly greater than that in the TD group (*β* = 0.33, *SE* = 0.07, *t* = 4.94, *p* < 0.001).

#### 3.2.3 Intensity of post-focus verbs

**Figure 11:**
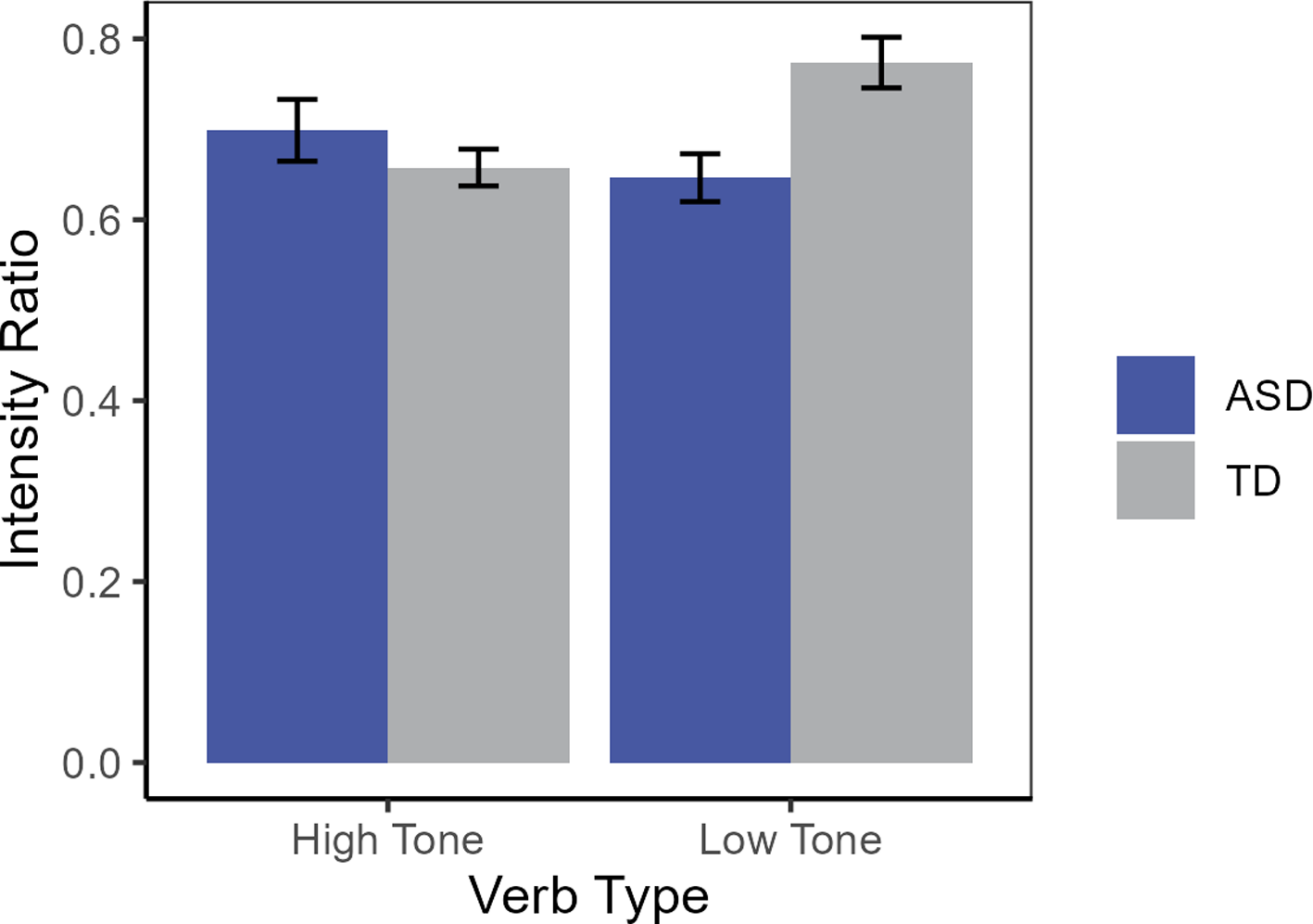
Intensity patterns of post-focus verbs in children with ASD and TD children.

For the intensity of post-focus verbs (see Figure 11), the results showed no significant main effects (all *p*s > 0.05) but a significant interaction between “Group” and “Verb Type” [*χ*^2^ (1) = 12.38, *p* < 0.001]. In addition, working memory, language level, and nonverbal intelligence did not show significant effects on the intensity ratio of verbs following sentence-initial focus (all *p*s > 0.05).

Pairwise comparisons on the interaction between “Group” and “Verb Type” showed that, for high-tone verbs, the difference in post-focus verb intensity ratio between the ASD and TD groups was not significant (*β* = −0.01, *SE* = 0.08, *t* = −0.14, *p* = 0.892); for low-tone verbs, however, the intensity ratio of the ASD group was significantly lower than that of the TD group (*β* = −0.18, *SE* = 0.09, *t* = −2.08, *p* = 0.042).

## 4 Discussion

This study thoroughly examined the prosodic characteristics of contrastive focus in Mandarin-speaking children with ASD, focusing on pitch, duration, and intensity differences between sentence-initial and sentence-final focus. Our findings demonstrate that prosodic focus marking in children with ASD is strongly influenced by focus position and interacts with lexical tone and post-lexical prosody, revealing a nuanced profile of prosodic processing in this population.

Under the sentence-initial focus condition, the ASD group showed significantly lower intensity on focus words compared to the TD group, regardless of verb tone. This pattern indicates that children with ASD produced less robust intensity modulation when marking focus on the subject noun. In addition, the ASD group showed a significantly smaller focus duration ratio than the TD group when the verb carried a high tone. These findings suggest that children with ASD have particular difficulty in marking sentence-initial focus, especially in the intensity domain. Notably, in the post-focus region (i.e., the verb following the focused subject), the ASD group showed significantly higher pitch than the TD group, and for low-tone verbs, significantly longer duration and lower intensity. These patterns indicate that children with ASD produced less acoustic distinction between focused and non-focused material in the sentence, with reduced pitch lowering, less duration reduction, and lower intensity in the post-focus region compared to TD children. In a study of Cantonese-speaking children with ASD, Chen et al. (2025) similarly found an over-realization pattern in tone production, suggesting that children with ASD may prioritize lexical-tone accuracy over prosodic focus marking. This interpretation is consistent with the present findings and suggests possible difficulty in allocating cognitive resources when lexical tone and prosodic focus must be processed simultaneously.

Under the sentence-final focus condition, the two groups showed a pattern opposite to that observed under sentence-initial focus. The pitch of sentence-final focus words in the ASD group was significantly higher than that in the TD group, and for high-tone verbs, the ASD group also showed significantly higher intensity. This finding is consistent with studies reporting higher pitch in children with ASD (Chen et al., 2022), as well as research documenting difficulties in prosodic focus marking and discourse-final processing among individuals with ASD (Shriberg et al., 2001; Peppé et al., 2007; Bonneh et al., 2011). The present study further suggests that the relatively stronger performance of children with ASD in sentence-final focus may reflect a compensatory strategy rather than a genuine prosodic advantage. When focus occurs at the end of a sentence, children with ASD are not required to manage post-focus modulation, as no linguistic material follows the focused element; consequently, their limited prosodic processing resources can be concentrated on the focus word itself. This interpretation is consistent with recent evidence indicating that children with ASD show a stronger preference for focus expansion than for post-focus modulation (Chen et al., 2025), and that sung-speech training enhances focus expansion more effectively than post-focus modulation (Zhang et al., 2025). Taken together, these findings suggest that the core difficulty for children with ASD may not lie in enhancing focus information per se, but rather in suppressing non-focus information. Our findings indicate that Mandarin-speaking children with ASD are not simply globally impaired in prosodic focus marking. Rather, they show a specific processing pattern: they can enhance focus words through focus expansion but have clear difficulty with post-focus compression. Under sentence-initial focus, this pattern appears as insufficient post-focus compression and reduced overall prominence; under sentence-final focus, where post-focus compression is not required, focus marking appears relatively more intact.

The study also shows that individual-difference factors such as working memory, language level, and nonverbal intelligence did not significantly affect the prosodic characteristics of focus words. This suggests that some features of focus-prosody performance in children with ASD may be related to core symptoms such as social-communication difficulties and may be partly independent of general cognitive or language ability (Sharda et al., 2010; McCann and Peppé, 2003; Diehl et al., 2015). A deeper understanding of the correspondence between speech-prosody characteristics and different symptom dimensions of ASD may contribute to theoretical models of autism and to more targeted clinical and educational support.

However, nonverbal intelligence did significantly predict the pitch of post-focus verbs. This partial finding is interesting in light of the meta-analytic result that nonverbal IQ and expressive language skills are significant moderators of prosodic focus production accuracy (Kuang et al., 2026). The meta-analysis included a much larger sample and broader range of cognitive abilities, whereas our study focused on a relatively narrow band of cognitive functioning. The discrepancy suggests that individual differences may become more apparent when samples include a wider range of cognitive profiles, and that post-focus compression may be particularly sensitive to nonverbal cognitive abilities.

Several limitations of the present study should be acknowledged, which also point to important directions for future research. Firstly, our sample size (20 children with ASD and 20 TD children), while comparable to many studies in this field, limits statistical power for detecting higher-order interactions and for fully examining the role of individual differences. Although we assessed working memory, language ability, and nonverbal intelligence and entered them as covariates in our statistical models, we acknowledge that the relatively modest sample size may limit the reliability of covariate adjustment. The fact that group differences in prosodic focus marking remained significant after covariate adjustment suggests that the observed prosodic impairments are not merely a byproduct of broader cognitive or language delays. However, this finding should be interpreted with caution, and we recognize that statistical control cannot fully eliminate confounding in small-sample group comparisons. Future studies with larger and more cognitively diverse samples are needed to further disentangle the relationships among language, cognition, and prosodic ability in ASD.

Secondly, although the present study provides detailed acoustic evidence for atypical prosodic focus production in children with ASD, it remains an open question whether these acoustic differences translate into communicative deficits, namely, whether listeners are actually less able to identify the intended focus when hearing speech produced by children with ASD. While our acoustic measurements demonstrate that children with ASD produce less robust prosodic cues to focus (e.g., reduced duration and intensity for sentence-initial focus, and weaker acoustic modulation in the post-focus region), we did not directly assess the perceptual consequences of these atypical productions. Future studies incorporating subjective listening tasks (e.g., forced-choice identification or rating tasks) alongside acoustic measurement would be valuable to determine whether the reduced prosodic modulation observed in ASD speakers is sufficient to impair listener comprehension in real-time communication. Such work would help bridge the gap between acoustic descriptions of atypical prosody and its functional, communicative impact.

Thirdly, we acknowledge that our design did not include a neutral-focus baseline condition, which precludes direct quantification of post-focus compression as conventionally defined (Liu and Xu, 2005; Xu, 1999). While our findings indicate that children with ASD showed reduced acoustic modulation in the post-focus region compared to TD children, we cannot directly attribute this pattern to impaired PFC per se. Future studies incorporating a neutral baseline are needed to directly quantify compression effects in ASD.

Moreover, our acoustic analysis focused on pitch, duration, and intensity—the three primary cues to prosodic prominence. However, other acoustic features, such as voice quality, spectral tilt, and fine-grained temporal alignment, may also contribute to focus marking and may be atypical in ASD. Future studies employing more comprehensive acoustic analyses (e.g., spectral measures, voice quality parameters) could provide a fuller picture of prosodic focus production in ASD.

Furthermore, our study was cross-sectional, which precludes conclusions about developmental trajectories. It remains unknown whether the difficulties in post-focus compression observed in school-aged children with ASD persist into adolescence and adulthood, or whether they diminish with age and intervention. Longitudinal studies tracking prosodic focus development from early childhood through adolescence would help answer this question and inform the optimal timing for clinical intervention.

In addition, a limitation of the present design is that the high-tone and low-tone verbs were based on two different syllables (*chī* and *xǐ*), which differ segmentally. Thus, the comparison between verb tones may be confounded by inherent phonetic and lexical differences. Although participants and items were included as random effects in the models to reduce item-specific biases, this approach cannot fully eliminate such systematic confounds. Future studies could address this limitation by using the same segmental carrier across different tonal conditions (e.g., mā/má/mǎ/mà) or by including a larger and more balanced set of verbs across tone categories to better isolate tonal effects from segmental influences.

Finally, the generalization of our findings to other tone languages requires further investigation. While Mandarin and Cantonese are both tone languages, they differ in tonal inventory and phonetic realization. Future cross-linguistic studies comparing Mandarin-speaking and Cantonese-speaking children with ASD would help determine whether the interaction between lexical tone and focus prosody observed here reflects a universal feature of tone-language processing in ASD or is specific to Mandarin.

## 5 Conclusion

This study demonstrates that Mandarin-speaking children with ASD use pitch, duration, and intensity as prosodic cues to signal contrastive focus, and that their use of these cues varies depending on focus position and tonal context. While they can enhance focus words, particularly in sentence-final position, they show reduced duration and intensity in sentence-initial focus and atypical acoustic patterns in the post-focus region. These findings suggest that the main challenge for children with ASD may not be the production of prosodic prominence per se but rather the modulation of surrounding non-focal elements. Given Mandarin’s requirement for simultaneous management of lexical tone and focus-related pitch, these results underscore the importance of investigating prosody in tonal languages within ASD research. Future work should examine larger samples, incorporate perceptual validation, and utilize longitudinal or intervention designs to track development and optimize strategies for supporting prosodic-focus abilities in clinical and educational settings, ultimately improving communication outcomes for children with ASD.

## Data Availability

The data supporting the conclusions of this article can be made available upon request. Requests to access these datasets should be directed to jinting0107@126.com.
